# ANALYSIS OF THE PROBLEM OF PSYCHOTHERAPY OF MENTAL HEALTH AND ADMINISTRATION IN THE AREA OF BIOETHICS

**Published:** 2016-12-30

**Authors:** Márquez Mendoza O., Fernández-Carrión M-H., Veyta López M., M.V. Flores Merino

**Affiliations:** 1 Head of the Research Group of Bioethics and Mental Health of the Research Center in Medical Science (CICMED) of the Autonomous University of the Satate of Mexico (UAEMex); 2 Head of the Research Center of Comparative Studies in Latin America; 3 Head of the Research Group of Biomedical Research, CICMED, UAEMéx; 4 Researcher, CICMED, UAEMex

**Keywords:** Bioethics, Mental health, Psychotherapy, Public administration

## Abstract

This research refers to the case study of a patient, a male college student, who was given psychotherapeutic care. Initially, the patient self-referral to the Mental Health area, which is a service permanently offered by the Autonomous University of the State of Mexico, through the Research Center in Medical Sciences (CICMED). The process of the case allowed analyzing the administrative procedure that operates in the CICMED and the process between this and the educational establishment in which the patient studied.

## INTRODUCTION

Two key elements stand out in this study: first, there is a scientific and technological advance that places us on the frontier of knowledge of bioethics in fields such as: genetic engineering and assisted reproduction. In contrast, we find that there is all kind of expressions of violence, cruelty and destruction, in the different spheres and levels of life, whose waves are increasing. Paradoxically; progress of societies show pulsional duality, described by Sigmund Freud in the psychoanalytic theory, that expresses the dimension of the human being as the most wonderful and monstrous being that exists in nature (Braunstein, 1981: 194). In this last point, according to Erich Fromm, aggression in people can not only be explained in terms of animal inheritance or destructive instinct. In fact, it must be understood on the basis of those environmental factors that act on the biological. All this raises a consideration of three aspects: 1) the practice of mental health institutions, both public and private, since they display administrative processes that can be evaluated and improved, 2) the bioethical responsibility of higher education institutions to address mental health problems in their student community and 3) the family and their bioethical response to mental health problems of their family members. Efforts were made to provide elements of improvement of the administrative process in the CICMED, in order to generate a more efficient care system for its patients. On the other hand, attempts were made to overcome the mind-body dualism, by linking not only the organic, psychological and social aspects but also to include all bioethical aspects. The purpose was to promote an inclusive and interdisciplinary psychiatry - for a better understanding of the sick in their conscious and unconscious dimensión. Also, it is important the generation of bioethical reflections that guide the institutions of higher education, the professionals and the administrative personnel towards a more committed attitude from the moral philosophy. From the operational and application point of view, it is sought to think over this perspective, hoping that this will give a new perspective in the student support programs. Particularly, those related to mental health - that are currently applied in the Institutions of Higher Education (HEI). Finally; this vision, congruent with the psychoanalytic optics under the assumption that mental illness, as a human act, contains a sense - seeks precisely to contribute to the understanding of the patient and contribute to a more comprehensive and contemporary psychotherapy.

## MATERIALS AND METHODS

The case study was constructed from the clinical attention provided to a student of an educational institution of the university. The program in the mental health area of the CICMED, due to exogenous causes to the institute itself, was limited to four months. However, the follow-up of the case was maintained for two more months. Once it was considered that the patient probably would not return, we opted to initiate the present research study. It should be noted that, the case was referred to the CICMED when it showed an advanced drug abuse, aggressive behavior in classrooms, recurrent absences in class sessions and difficulties in family dynamics. This reveals the importance of the creation of assessment systems to analyze and evaluate the follow-up of atypical cases in each establishment of Higher Education Institutions (HEIs), with full involvement of instances such as CICMED. As well as specialized areas in the diagnosis of behavioral cases of this nature. It is recommended that each HEIs consider to design and to operate a diagnostic program, also the referral of limiting situations, which would generate a more efficient, effective and timely clinical-administrative system. Then, in order to provide elements of judgment to identify the basic characteristics of the case, a general summary is presented and, in order to fully preserve the identity of the patient, he has become known as Pedro B.

### Synopsis of the case:

characteristics of the patient: name: Pedro B.; Age: 20 years old; male; Marital status: single; occupation: student; academic year: first (second semester), bachelor degree in a public institution.

### Admission and evolution of the case:

Pedro B. was a student of an educational establishment that was part of an institution of higher education. He was self referral in the area of Mental Health in the CICMED, on November 5, 2001. The purpose of his visit was to request psychological attention due to behavior problems in the school (The administrative and clinical process that traced the case is represented in the Gantt diagram).

During the evaluation process, the following symptoms were observed: perplexity, isolation, anxiety, alterations of language and communication; derailment or laxity of associations, tangentiality, neologisms and disorders of thought content (Spitzer, 2000). Therefore, a combined psychopharmacological and psychotherapeutic treatment based on Olanzapine (Olanzapine: major tranquilizer with minimal side effects versus other drugs of the same type; Ziprexa tablets 10 mg/night) was proposed. A weekly consultation program was established, in order to maintain a permanent assessment and a monitoring process. Initially, it was suggested to the patient to refer to a hospital care and, if necessary, hospitalization facilities, since the CICMED only offers external consultation. However, the patient insisted to be attended by a specialist of the university, as he had been attended at university from the beginning. This indicated an expression of positive transference by the patient and the family, which would be solved and clarified during the therapeutic process. Likewise, the counter transference of the therapist in the same positive way towards the patient and his family. The first interviews were made by explaining the following points: it would be permanently informed of the evolution that would occur, as well as the crises of both the family structure and the patient. The prudence and control would be manifested continuously at all times inside and outside the consultation. Therefore, the progress made would be register with the purpose to alleviate suffering, the degree of recovery of family functioning, improvement in the psychotic symptoms of the patient, the elaboration of the grief of childhood, the degree of awareness of his person and his dignity, as well as the possibilities to contiue with school activities.

Once expressed these points and derived from the attitude of human commitment established previously, it was decided to sustain this demand for care, consistent with the medical ethics (Velasco, 1998: 55) and the psychoanalysis (Lacan, 1981: 307). The frame initially proposed consisted of one session per week on Monday at 11:30 am, which began on November 5, 2001 and ended in the second week of February 2002. The development of the same can be observed in the Gantt diagram. In this case, the educational unit was informed of the evolution of the patient and his assistance through a reference-counter-reference note. The authorities of the educational establishment were informed that if the patient did not attend regularly to the psychotherapeutic treatment, he could not attend any school activity, due to the clinical frame. The educational establishment was notified of the situation in the case. Then, it was explained to the family and the patient the importance of maintaining adequate treatment, and that depending on the results and possible evolution, he could return to their school activities. However, after some attendance and multiple absences to therapy the patient expressed returning to the treatment indicated by the CICMED. Specially, after a fight with several of his colleagues, and from the second fortnight of January and practically the first two weeks of February 2002. As a result of a new assessment, the Mental Health specialist at the institute verified that the patient continued with the problem of drug addiction (drug dependence in Mexico is analyzed by Medina-Mora, 2002; the use of toxic substances may cause transient psychosis) and recommended an institution specializing in addiction treatment, since it is a patient with probable psychosis (Freud, 1982: XVI, 224) (see Gantt diagram).

### Problem statement

Based on the above, a series of considerations can be projected around the bioethical role. On one side, the responsibility of the educational institution (Autonomous State University from Mexico) and, on the other hand, the body responsible for attending the cases referred to the institution itself (Center for Research in Medical Sciences) for its clinical and psychiatric care. Finally, the role of the family in the described case. The response to these approaches will allow us to reach the following objectives: 1. To support the generation of proposals that contribute, from a bioethical approach, to the strengthening of the strategies carried out, in the area of prevention and treatment of mental health. This is important for the family and in educational health institutions (public or private), 2. To promote the review and critical analysis of administrative systems of health entities, based on general conditions of a case and seeking its possible application in other similar conditions. 3. To open a space for reflection that seeks the interrelation among the clinical, administrative and bioethical scope.

## RESULTS AN DISCUSSION

It is important to emphasize that for this study, the authorization of the patient and his family was required. The following bioethical principles were applied (proposed by Tom L. Beauchamp and James F. Childress in Principles of Biomedical Ethics, 1979, and in this study case based on the criterion established by Jonsen 1994): 1. Principle of informed consent, which establishes the freedom of information 2. Basic principles of respect for people, from the ethical and legal point of view both verbally and in writing. 3. Principle of nonmaleficence, which formulate two complementary rules: a) do not harm and b) maximize potential benefits and minimize potential risks. 4. Principle of justice, evaluates who should receive the benefit of the research, and if it is exempted from any suffering for applying the anonymity of the research subjects favoring the care of mentally ill patients. Informed consent is always verbal and in written. It ensures that the most relevant information has been given by the physician and it is received by the patient and his / her family. To consider this autonomy, it is required the willfulness. Any consent, in which information has been issued by an ill patient who does not act on a voluntary basis, it is not acceptable from the ethical and legal point of view.

### Conclusion

This case presents, with great clarity, the disarticulation existing between the medical and administrative (internal and external) instances that affected the patient in iatropatogenic form. Therefore, it is important the creation of a Bioethics Committee within the medical institutions of higher education. The committee can attend this type of cases and guarantees in an objective and impartial manner the human rights of the community with its different actors (students, teachers and administrators). Also, it advises educational institutions on how to address and solve this type of problem. The support of the previous proposal is based on the principle of bioethics as a new discipline that combines biological, psychological and social knowledge with the system of human values. This allows us to speak of a new intellectual paradigm consisting of confrontation between facts and values. Similarly it is convenient to carry out the following strategies within the institutional framework to positively influence a change in the autonomy of people to decide on mental health problems: 1. To develop continuing education programs in mental health, aimed to counselling and teaching staff who are in direct contact with the student community.2. To strengthen the programs of continuing education in the area of mental health, directed to the personnel dedicated to this task in the public institutions. 3. To increase the research in the field of mental health, particularly in the area of prevention.

## Figures and Tables

**Table 1.** Gantt diagram of psychotherapy applied to Pedro B. in the CICMED

N.	Activities	2001–2002								
		N.		D.		E.			F.	
1	Pedro B. referral for clinical evaluation									
2	Diagnostic application									
3	Prescription of treatment									
4	Appointment next week. Improvement									
5	Two weeks absence									
6	The mother of Pedro B. request an excuse absence of school for the patient									
7	Request of presence of the patient									
8	The patient is absent 40 days									
9	Patient hospitalization in a psychiatric clinic for 40 days									
10	The mother of Pedro B .explains that he was hospitalized									
11	The contact with the educational department is established									
12	A therapeutic contract is generated to continue with the prescribed treatment. Family and patient									
13	The patient gets back to the treatment									
14	Official suspension of Pedro B. by the school authority									
15	Official referral of the patient by the educational establishment									
16	A general report is given to the educational establishment									
17	The patient continues with the sessions and the treatment									
18	Second assessment of the patient									
19	It is concluded that the patient continues with the use toxic substances									
20	Referral to an institution specializing in addictionis recommended									
21	Agreement with the family of the patient about the treatment he will receive									
22	Lack of information									

Source: own elaboration
